# ICU service in Taiwan

**DOI:** 10.1186/2052-0492-2-8

**Published:** 2014-02-05

**Authors:** Kuo-Chen Cheng, Chin-Li Lu, Yueh-Chih Chung, Mei-Chen Huang, Hsiu-Nien Shen, Hsing-Min Chen, Haibo Zhang

**Affiliations:** Department of Internal Medicine, Chi Mei Medical Center, Tainan, Taiwan; Department of Safety Health and Environmental Engineering, Chung Hwa University of Medical Technology, Tainan, Taiwan; Department of Medicine, National Defense Medical Center, Taipei, Taiwan; Department of Medical Research, Chi Mei Medical Center, Tainan, Taiwan; Bureau of National Health Insurance Kao-Ping Branch, Kaohsiung, Taiwan; Department of Intensive Care Medicine, Chi Mei Medical Center, Tainan, Taiwan; The Keenan Research Centre in Biomedical Science, St. Michael’s Hospital, Toronto, University of Toronto, Room 619, 209 Victoria Street, Toronto, Ontario M5B 1T8 Canada

**Keywords:** Medical expenditure, ICU structure, Bed occupancy, Critical illness

## Abstract

**Background:**

The aim of the study was to understand the current status of intensive care unit (ICU) in order to optimize the resources achieving the best possible care.

**Methods:**

The study analyzed the status of ICU settings based on the Taiwan National Health Insurance database between March 2004 and February 2009.

**Results:**

A total of 1,028,364 ICU patients were identified. The age was 65 ± 18 years, and 61% of the patients were male. The total ICU bed occupancy rate was 83.8% which went up to 87.3% during winter. The ICU bed occupancy was 94.4% in major medical centers. The ICU stay was 6.5 ± 0.5 days, and the overall ICU mortality rate was 20.2%. The hospital stay was 16.4 ± 16.8 days, and the average cost of total hospital stay was approximately US$5,186 per patient.

**Conclusions:**

The rate of ICU bed occupancy was dependent on seasonal changes, and it reached near full capacity in major medical centers in Taiwan. The ICU beds were distributed based on the categories of hospitals in order to achieve a reasonable cost efficiency. ICU faces many challenges to maintain and improve quality care because of the increasing cost of state-of-the-art technologies and dealing with aging population.

## Background

The intensive care unit (ICU) is a specialized department in a hospital that provides extensive care for critically ill patients. The ICU has grown rapidly in hospital systems throughout the past decade [1, 2]. The high demand of staffing, the requirement of state-of-the-art equipment, and the increased complexity of diseases all raise concern for cost efficiency in the ICU [1]. A great deal of differences in ICU expenditure has been noted across the globe [3]; reducing or controlling the costs while maintaining and improving the quality of ICU is urgently needed not only by administrators and policy makers but also by practicing physicians [4].

The National Health Insurance (NHI) system in Taiwan has been successful during the past decade in maintaining its stable and relatively low costs while keeping high satisfaction of its policy coverage [5]. The national health expenditure increased at a modest rate from 5.1% of gross domestic product (GDP), when the NHI was introduced in 1995, to 6.2% in 2005 [5–7].

In the present study, we report an overall analysis by extracting the NHI database on the utilization of ICU resources in relation to outcomes of critically ill patients in Taiwan during the period of March 2004 to February 2009.

## Methods

### Database and study design

This study was approved by the institutional Research Ethical Board of Chi Mei Medical Center. An informed consent form was not required given the nature of the administrative purpose of the NHI database. A total of 1,028,364 adult patients (≥18 years) hospitalized at ICU between March 2004 and February 2009 were included for analysis.

The NHI system has been compulsory for providing universal coverage to all citizens, with the exception of prisoners, since 1995. The NHI database covers a population of approximately 22 million [8] and provides encrypted information regarding patients’ identification, gender, date of birth, dates of hospital admission and discharge, medical institutions that provided the services, the International Classification of Diseases (ninth revision), the Clinical Modification (ICD-9-CM) codes for diagnosis (one principal and four subordinate diagnoses), outcome at hospital discharge, and medical expenditures.

The case mix index (CMI), which is a reflection of the cost of caring and utilization of resources, was calculated based on the methods of diagnosis-related groups, edition 3.2 [9]. The Charlson comorbidity index (CCI) was determined for the evaluation of the severity of diseases [10–12].

### Three categories of hospital systems

The health care system in Taiwan consists of three categories of clinical organizations: (1) the local clinics for outpatients,(2) the district hospitals (DH) and regional hospitals (RH), and (3) the large medical centers (MC). The system is regularly evaluated based on the number of beds and employees, the resource of major equipment for diagnosis and treatment, and the quality of care by a State Joint Commission on Hospital Accreditation. Generally speaking, the number of acute beds is over 500 in MC, over 250 in RH, and less than 250 in DH; the quality of care is higher in MC, followed by RH and DH [13].

### Term definitions and measurements

ICU bed occupancy rate was calculated by the number of occupied ICU bed per day divided by the number of available ICU beds multiplied by the number of days in a given year. The average ICU stay was calculated by dividing the total number of days that ICU patients stayed over the number of admissions in the given year. Traditionally, some Taiwanese patients became accustomed to requesting for discharge at the end stages of diseases in order to decease at home rather than in a hospital. Thus, ICU mortality included the patients who died at ICU and those who were discharged from ICU for decease at home.

Once the primary diagnoses of diseases were identified, the incidences of the diseases were ranked as the following: (1) ischemic heart diseases (ICD-9CM 410–411), (2) pneumonia (ICD-9CM 480–488), (3) stroke (ICD-9CM 430–438), (4) acute respiratory failure (ICD-9CM 518.81,518.82,518.84), (5) septicemia (ICD-9CM 038.0–038.9), (6) heart failure (ICD-9CM 428.0–428.9), (7) chronic obstructive pulmonary disease (COPD; ICD-9CM 490–496), (8) liver cirrhosis (ICD-9CM 571), (9) urinary tract infection (ICD-9CM 599.0), and (10) shock without documentation of trauma (ICD-9CM 785.50–785.52, 785.59).

The analysis was performed for the utilization of resources including diagnostic and therapeutic procedures such as computerized tomography (CT), magnetic resonance imaging (MRI), mechanical ventilator (MV), noninvasive positive pressure ventilator (NIPPV), extracorporeal membrane oxygenation (ECMO), and intra-aortic balloon pump (IABP).

The four seasons were defined as spring (March to May), summer (June to August), fall (September to November), and winter (December of a given year to February in the following year).

### Statistical analyses

Data analyses were performed using SPSS for Windows, version 17.0 (Chicago, IL, USA). Data are reported as mean ± standard deviation (SD) or median and interquartile range (IQR). Patient distribution and primary diagnoses across the three categories of hospitals were analyzed using a chi-square test; the trend of analysis of variables including gender distribution, mechanical ventilator weaning rate, and ICU mortality was performed using Mantel-Haenszel linear-by-linear test. Differences were analyzed using ANOVA.

Medical expenditure was standardized using the Consumer Price Index in 2009 as a reference level. The values of cost are reported in US dollars after conversion from Taiwan dollars based on an average exchange rate as of 2009.

## Results and discussion

### Results

#### Demographic characteristics

The numbers of beds in ICU and in hospitals increased from 2004 to 2008. The ratio of ICU beds per 100,000 inhabitants increased from 28.9 in 2004 to 30.4 in 2008 (Table 1).

**Table 1:** **The ratio and number of beds in ICU and hospitals**

Year	ICU beds/hospitals beds				Ratio ^a^ of ICU beds/100,000 population
	MC	RH	DH	Total	
2004	2,417/31,195(7.75%)	2,902/43,628(6.65%)	1,233/35,952(3.43%)	6,552/110,775(5.91%)	28.87
2005	2,391/30,552(7.83%)	3,045/43,021(7.08%)	1,360/38,584(3.52%)	6,796/112,157(6.06%)	29.85
2006	2,454/31,786(7.72%)	3,055/37,616(8.12%)	1,405/44,800(3.14%)	6,914/114,202(6.05%)	30.22
2007	2,549/32,439(7.86%)	2,990/30,949(9.60%)	1,441/52,937(2.72%)	6,980/116,325(6.00%)	30.40
2008	2,588/33,462(7.73%)	3,118/35,435(8.80%)	1,303/49,739(2.62%)	7,009/118,636(5.91%)	30.42

*MC* medical centers, *RH* regional hospitals, *DH* district hospitals. ^a^Numbers of population per year were obtained from the Department of Statistics, Ministry of the Interior, Taiwan.

A total of 1,028,364 patients were admitted to the ICU, and 626,634 (60.9%) of the patients were males aging 65 ± 18 years. The number of patients admitted to ICU increased from 192,858 in 2004 to 213,030 in 2008, and the age rose from 64.5 years in 2004 to 65.6 years in 2008 (*P* < 0.001, Table 2).

**Table 2:** **Demographic characteristics of the study population**

		Number	Age (year)	Male (%)	CMI	CCI
			Mean ± SD		Mean ± SD	Mean ± SD
						Median (IQR)
Year	2004	192,858	64.5 ± 18.0	60.8	2.12 ± 1.89	1.68 ± 1.91
						1 (0–2)
	2005	202,788	64.7 ± 18.0	61.1	2.11 ± 1.85	1.65 ± 1.90
						1 (0–2)
	2006	207,528	64.9 ± 18.0	61.0	2.14 ± 1.91	1.68 ± 1.93
						1 (0–2)
	2007	212,160	65.4 ± 17.9	60.6	2.23 ± 1.97	1.68 ± 1.91
						1 (0–2)
	2008	213,030	65.6 ± 17.8	61.2	2.29 ± 2.01	1.69 ± 1.92
						1 (0–2)
*P* value for trend			<0.001	0.486	<0.001	0.025
Hospital	MC	354,631	61.8 ± 18.2*	61.6*	2.71 ± 2.54*	1.70 ± 2.08**
						1 (0–2)
	RH	488,683	65.4 ± 17.7**	60.9**	2.02 ± 1.61**	1.70 ± 1.90**
						1 (0–2)
	DH	185,050	70.4 ± 16.7	59.9	1.71 ± 1.15	1.57 ± 1.60
						1 (0–2)
Total		1,028,364	65.0 ± 18.0	60.9	2.18 ± 1.93	1.68 ± 1.91
						1 (0–2)

*MC* medical centers, *RH* regional hospitals, *DH* district hospitals, *SD* standard deviation. *IQR*, interquartile range, *CMI* case mix index, *CCI* Charlson comorbidity index. *P < 0.001 vs. RH and DH; **P < 0.001 vs. DH.

The number of ICU patients admitted to MC was 354,631 (34.5%), 488,683 (47.5%) to RH, and 185,050 (18%) to DH. The CMI value significantly increased from 2.1 in 2004 to 2.3 in 2008 (*P* < 0.001 for trend analysis) and was significantly higher in MC (2.7 ± 2.5), followed by RH (2.0 ± 1.6), and DH (1.7 ± 1.2) (*P* < 0.001, MC vs. RH and DH, and RH vs. DH, respectively). The overall CCI value remained almost identical over the study period but was significantly higher in MC and RH than in DH (*P* < 0.001, Table 2).

#### Total ICU bed occupancy

**Figure 1:**
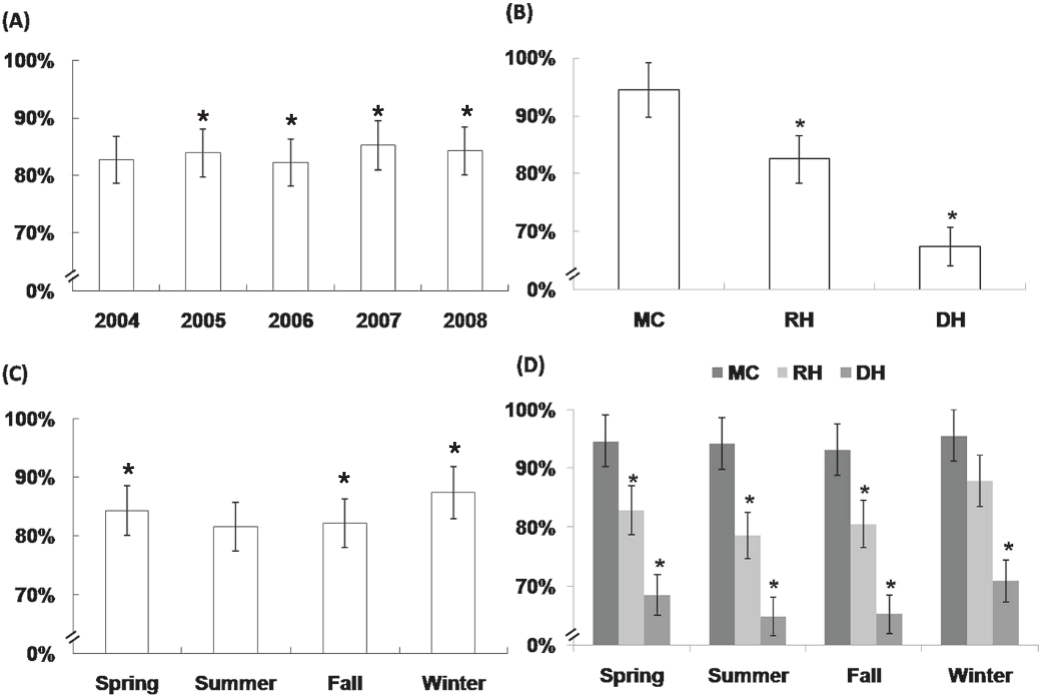
**Total occupancy rate of ICU beds.** They are by **(A)** calendar years, **(B)** different categories of hospitals, **(C)** seasons, and **(D)** seasonal variations of different hospitals. *MC* medical centers, *RH* regional hospitals, *DH* district hospitals. *P < 0.05 compared to that in 2004 in A, compared to summer in C, and compared to MC in B and D, respectively.

The total occupancy rate of ICU beds increased over the study years from 82.7% in 2004 to 85.2% in 2007, and 84.2% in 2008 (Figure 1A). The average occupancy rate of ICU beds was 83.8%, with the highest of 94.4% in MC, followed by 82.5% in RH, and the lowest is at 67.4% in DH (Figure 1B).

#### Seasonal variations

The overall occupancy rate of ICU beds was highest (87.3%) in winter, followed by 84.3% in spring, 82.2% in fall, and was lowest (81.5%) in summer (Figure 1C). This pattern of ICU occupancy was also observed in the three categories of hospitals with the highest (95.6%) in MC, followed by 87.8% in RH, and 70.9% in DH during the winter season (Figure 1D).

Among the top ten primary diagnoses, the incidences of pneumonia, stroke, acute respiratory failure, heart failure, and liver cirrhosis were higher during winter; septicemia and urinary tract infections were more common during summer; and COPD was more dominant during spring (Table 3).

**Table 3:** **Seasonal variation of the top ten primary diagnoses in ICU from 2004 to 2008**

Primary diagnosis	Season				***P*** value	Total
	Spring	Summer	Fall	Winter		
IHD	32,518 (12.7%)	31,099 (12.3%)	31,977 (12.5%)	32,712 (12.5%)	0.001	128,306 (12.5%)
Pneumonia	22,699 (8.8%)	21,655 (8.5%)	20,394 (8.0%)	23,596 (9.0%)	<0.001	88,344 (8.6%)
Stroke	19,587 (7.6%)	18,098 (7.1%)	19,447 (7.6%)	22,104 (8.4%)	<0.001	79,236 (7.7%)
ARF	15,651 (6.1%)	14,916 (5.9%)	15,013 (5.9%)	17,197 (6.6%)	<0.001	62,777 (6.1%)
Septicemia	12,225 (4.8%)	13,822 (5.5%)	13,568 (5.3%)	13,000 (5.0%)	<0.001	52,615 (5.1%)
Heart failure	6,675 (2.6%)	5,473 (2.2%)	6,320 (2.5%)	7,578 (2.9%)	<0.001	26,046 (2.5%)
COPD	7,005 (2.7%)	6,290 (2.5%)	5,888 (2.3%)	6,505 (2.5%)	<0.001	25,688 (2.5%)
Liver cirrhosis	4,232 (1.6%)	4,081 (1.6%)	4,338 (1.7%)	4,631 (1.8%)	<0.001	17,282 (1.7%)
UTI	2,993 (1.2%)	3,411 (1.3%)	3,171 (1.2%)	2,824 (1.1%)	<0.001	12,399 (1.2%)
Shock	3,003 (1.2%)	2,928 (1.2%)	2,754 (1.1%)	2,740 (1.0%)	<0.001	11,425 (1.1%)

*IHD* ischemic heart disease, *ARF* acute respiratory failure, *COPD* chronic obstructive pulmonary disease, *UTI* urinary tract infection.

#### Utility of major equipment, outcomes, and expenditures

The frequency of using MRI, NIPPV, ECMO, and IABP slightly increased, while the use of CT and MV decreased from 2004 through 2008. The frequency of utilization of MRI, NIPPV, ECMO, IABP, and MV was greater in MC followed by RH, and then DH with an exception that the use of CT was almost identical in MC and RH.

The mean ICU days tended to decrease from 6.6 in 2004 to 6.3 in 2008, and the mean hospital days tended to decrease from 16.7 in 2004 to 16.1 in 2008. The mean mechanical ventilator weaning rate increased from 58.1% in 2004 to 64.2% in 2008. The overall mortality rate at the ICU was 17.7% in MC, 20.8% in RH, and 25.6% in DH. The mortality rate tended to decrease from 21.5% in 2004 to 19.0% in 2008, while the average expenses of hospitalization increased from US$5,051 in 2004 to US$5,480 (8.5%) in 2008 (Table 4).

**Table 4:** **ICU days, hospital days, ICU mortality, and total hospital cost**

			ICU days	Hospital days	Total hospital cost	ICU mortality (%)
Years	2004	Mean ± SD	6.6 ± 9.7	16.7 ± 17.5	5,051 ± 6,034	21.5
		Median (IQR)	3 (2–7)	12 (6–22)	3,313 (1,575–6,262)	
	2005	Mean ± SD	6.6 ± 9.3	16.5 ± 17.0	5,072 ± 6,003	20.5
		Median (IQR)	3 (2–7)	12 (6–21)	3,359 (1,593–6,275)	
	2006	Mean ± SD	6.5 ± 9.2	16.5 ± 17.5	5,129 ± 6,332	20.0
		Median (IQR)	3 (2–7)	11 (6–21)	3,388 (1,625–6,251)	
	2007	Mean ± SD	6.4 ± 9.0	16.2 ± 16.3	5,180 ± 6,066	19.9
		Median (IQR)	3 (2–7)	12 (6–21)	3,521 (1,701–6,397)	
	2008	Mean ± SD	6.3 ± 9.0	16.1 ± 16.2	5,480 ± 6,460	19.0
		Median (IQR)	3 (2–7)	12 (6–21)		
	*P* for trend		<0.001	<0.001	<0.001	<0.001
Hospitals						
MC	2004	Mean ± SD	6.7 ± 9.8	18.3 ± 17.4	6,623 ± 7,119	18.6
		Median (IQR)	3 (2–7)	13 (7–24)	4,638 (2,593–8,235)	
	2005	Mean ± SD	6.6 ± 9.7	18.2 ± 17.5	6,663 ± 7,238	17.8
		Median (IQR)	3 (2–7)	13 (7–24)	4,627 (2,587–8,315)	
	2006	Mean ± SD	6.6 ± 9.8	18.1 ± 17.9	6,766 ± 7,855	17.5
		Median (IQR)	3 (2–7)	13 (7–24)	4,578 (2,588–8,306)	
	2007	Mean ± SD	6.5 ± 9.6	17.6 ± 16.8	6,845 ± 7,397	17.6
		Median (IQR)	3 (2–7)	13 (7–23)	4,820 (2,765–8,439)	
	2008	Mean ± SD	6.4 ± 9.5	17.6 ± 16.6	7,277 ± 7,850	16.9
		Median (IQR)	3 (2–7)	13 (7–23)	5,125 (3,009–8,869)	
	*P* for trend		<0.001	<0.001	<0.001	<0.001
RH	2004	Mean ± SD	6.4 ± 9.4	16.2 ± 17.3	4,631 ± 5,535	22.1
		Median (IQR)	3 (2–7)	11 (5–21)	3,023 (1,479–5,618)	
	2005	Mean ± SD	6.3 ± 8.8	15.6 ± 16.7	4,629 ± 5,378	21.1
		Median (IQR)	3 (2–7)	11 (5–20)	3,090 (1,515–5,655)	
	2006	Mean ± SD	6.1 ± 8.6	15.5 ± 16.9	4,565 ± 5,394	20.9
		Median (IQR)	3 (2–7)	11 (5–20)	3,058 (1,511–5,511)	
	2007	Mean ± SD	6.0 ± 8.3	15.3 ± 15.8	4,595 ± 5,280	20.4
		Median (IQR)	3 (2–7)	11 (5–20)	3,144 (1,547–5,612)	
	2008	Mean ± SD	6.0 ± 8.3	15.3 ± 15.9	4,795 ± 5,540	19.6
		Median (IQR)	3 (2–7)	11 (6–20)	3,336 (1,651–5,814)	
	*P* for trend		<0.001	<0.001	<0.001	<0.001
DH	2004	Mean ± SD	7.1 ± 10.0	15.3 ± 17.9	3,274 ± 4,142	27.2
		Median (IQR)	3 (2–8)	10 (5–20)	1,866 (899–4,004)	
	2005	Mean ± SD	7.1 ± 9.7	15.2 ± 16.9	3,352 ± 4,109	26.4
		Median (IQR)	3 (2–8)	10 (5–20)	2,014 (964–4,148)	
	2006	Mean ± SD	7.3 ± 9.5	15.6 ± 17.0	3,497 ± 4,374	24.7
		Median (IQR)	4 (2–9)	10 (5–20)	2,116 (1,012–4,223)	
	2007	Mean ± SD	7.3 ± 9.3	15.3 ± 15.7	3,420 ± 3,929	25.7
		Median (IQR)	4 (2–9)	11 (5–20)	2,162 (1,066–4,326)	
	2008	Mean ± SD	7.3 ± 9.5	15.5 ± 15.9	3,654 ± 4,501	23.9
		Median (IQR)	4 (2–9)	11 (5–21)	2,372 (1,167–4,594)	
	*P* for trend		<0.001	0.278	<0.001	<0.001
Total		Mean ± SD	6.5 ± 9.2	16.4 ± 16.8	5,186 ± 6,188	20.2
		Median (IQR)	3 (2–7)	12 (6–21)	3,479 (1,664–6,383)	

## Discussion

There are several interesting messages that are offered by the study: (1) we observed a trend of an increase in aging population in the ICU over the study period that led to a greater need for ICU beds [14], (2) the ICU beds were in greater demand in the academic MC than in RH and DH, (3) there was variance of ICU bed occupancy depending on seasonal changes, (4) CMI appeared to be a reliable indicator for consumption and severity of patient conditions, and (5) the incidences of pneumonia, stroke, heart failure, and liver cirrhosis were higher in winter than in other seasons, thus conceivably contributing to a high mortality as reported previously [15–17].

Our data suggests that the increasing demand of ICU beds was partially due to the progressively aging population over the study period in Taiwan. This trend may also be implicated in other countries although no such studies were found, to the best of our knowledge; indeed, the rise of the aging population is a general trend worldwide [18]. A recent report shows that the population over 65 years of age is 11% in Taiwan, 13% in the USA, 17% in both England and France, 21% in Germany, and 24% in Japan [18]. Taken together, there may be an urgent need in future strategic plans to adjust the ratio of ICU beds over hospital beds in order to better serve our aging societies.

It is noteworthy that although CMI has been widely used for the evaluation of medical resources consumed by individual hospitalized patient [19], CMI can also be used for the evaluation of the severity of critically ill patients [19, 20]. Indeed, we observed an increase in CMI over several years, in which the highest CMI was noted in MC where the sicker patients were treated, followed by RH and DH, suggesting that the consumption of resources is correlated with the severity of illness. The CCI value increased slightly over the study period but was significantly higher in MC and RH as compared with DH. It is compatible with the findings of CMI where more critical patients were managed in MC and RH as compared with DH.

The ICU bed occupancy is highest in MC, followed by RH and DH. This phenomenon might be explained by the fact that most patients preferred to be treated at the urban MC which usually offer better resources and quality care. The ICU bed occupancy rate observed in this study may be applicable to most Asian countries where the structure of health care systems is similar to that of Taiwan; however, to the best of our knowledge, no study was reported for such a comparison as demonstrated in this study.

We have observed that ICU bed occupancy increased during winter; our data is in agreement with that of Garfield and colleagues [21]. The investigators analyzed data on 16,355 critically ill patients who were admitted to ICUs in five adjacent hospitals in East Anglia between 1992 and 2000. They showed a 30% higher admission rate in December than the month of February. Taken together, it is clear that there is significant seasonal variation, and it is important to take this fact into account for planning ICU services.

The bed occupancy rate reflects not only hospitals’ efficiency but also the quality and provision of safety [22]. From our own experience, keeping the ICU bed occupancy at 80% to 85% is good enough to meet the requirements for new admission of critically ill patients. An average occupancy level of 82% is suggested to be of optimal range [23]. Hospitals with average occupancy rates above 85% are considered less efficient and therefore would likely increase the waiting time for emergency patients [22, 24], which may lead to increase in infections such as methicillin-resistant *Staphylococcus aureus*[25]. However, our data also suggests that the degree of quality care may also play an important role in compensation of a high occupancy rate with respect to mortality rate. This is supported by our data, which shows that although the ICU bed occupancy was high, the mortality rate was well controlled in the MC compared to other hospital categories.

Delayed ICU admission due to shortage of beds at first referral is associated with increased mortality rates on days 28 and 60 [26]. Significant correlation between time of admission and survival rates was also noted. Each hour of waiting was an independent factor in the 1.5% increase of the risk of ICU death [27].

The tightly controlled hospital expenses that was increased by 2.3% yearly was mainly due to the regulation of global budget system that applies an expenditure cap in all medical specialties, including critical care. If expenses exceeded the budget limit, the reimbursement/consumption ratio would be decreased for each service conducted [28, 29]. These circumstances would lead to cutting down the number of employees and hindering any purchase of new equipment or medications, thus potentially compromising quality care and patient safety [30, 31].

The ratio of ICU beds to the number of inhabitants appeared to have an impact on quality care in our study. Although the ratio of adult ICU beds was somewhat higher in Taiwan (29.8) than in the UK (3.3) and in Germany (24) per 100,000 population in 2005 [32, 33], it is impossible to compare the ICU mortality rate given the drastic differences in routine care and discharge policies among the countries [2, 34]. A previous study in North America and Europe showed an inverse correlation between ICU beds and hospital mortality [3].

It is difficult to compare the dollar value among countries due to different economic and consumption statuses as well as ICU service systems [35]. For example, the total health expenditure per capita in 2010 was US$8,233 in the USA, US$4,338 in Germany, US$3,974 in France, US$3,433 in the UK, US$3,204 in Japan, and only US$1,284 in Taiwan [32, 33]. The ratio of the total health expenditure per capita/GDP in 2010 was 17.3% in the USA, 10.7% in Germany, 9.4% in France, 9.6% in the UK, 7.8% in Japan, and 6.9% in Taiwan [36]. Since the ICU mortality rates were similar among the countries mentioned [1, 3, 6], it suggests that the expenditure could be reduced to maintain similar quality care.

There are several limitations in this study which include the lack of information of APACHE II scores and therapeutic intervention scoring system due to missing data. We were unable to obtain the exact ICU expenses because they were not separately reported in the NHI database, but it is important to understand the direct cost effectivity in ICU settings. These issues will be addressed in future data collection of the NHI system.

## Conclusions

In conclusion, the utilization of medical resources and the ICU outcomes are considered as reasonably cost-effective in Taiwan. The well-controlled ICU cost might be due to the limited length of ICU stay and the regulatory restriction of ICU expenditure gap by the insurance system. Many challenges are ahead with respect to maintaining and further improving the quality of ICU care, including the assurance of resources for state-of-the-art techniques and the aging population.
